# Implementation and outcomes of a digital onboarding taskforce in the acute care setting

**DOI:** 10.3389/fdgth.2025.1639828

**Published:** 2025-12-17

**Authors:** Julianna LeNoir, Alexzandra Gentsch, Akshay Krishnan, Jeffrey Ndubisi, Marissa Witmer, Kristin L. Rising, Brooke Worster, Angela M. Gerolamo

**Affiliations:** 1 Center for Connected Care, Thomas Jefferson University, Philadelphia, PA, United States; 2 Department of Emergency Medicine, Sidney Kimmel Medical College, Thomas Jefferson University, Philadelphia, PA, United States; 3 Division of Supportive Medicine, Department of Medical Oncology, Philadelphia, PA, United States; 4 School of Nursing, University of Pennsylvania, Philadelphia, PA, United States

**Keywords:** digital health, patient porta, telehealth, patient education, digital literacy, health technology access, patient-centered care

## Abstract

**Introduction:**

Use of digital health technology can improve patient health outcomes; however, not all patients have the knowledge and skills to download a health app and access a patient portal. Providing digital onboarding support to hospitalized patients has potential to overcome some barriers to accessing needed education in the community, including both having the time and a location to receive education. To address this, our team developed the Jefferson Digital Onboarding Taskforce (JeffDOT), a group of staff and students who approach hospitalized patients and provide one-on-one teaching on how to sign up for and use a patient portal.

**Methods and materials:**

This descriptive study examined the implementation and preliminary outcomes of JeffDOT. We collected patient demographics and assessed health literacy, digital health readiness, and empowerment using the patient portal after patients received individualized support with portal enrollment.

**Results:**

We enrolled 343 hospitalized patients from a large academic medical center in the U.S. in their patient portal. Almost half of the sample (49%) was older than 55 years, 56% were male, 34% were Black, and 19% spoke Spanish at home. After receiving individualized support from the JeffDOT team, the majority of patients (84%) reported that they felt empowered to use the portal and almost half reported that they would be very interested in additional basic computer skills training if offered by the hospital.

**Discussion:**

Our findings suggest that supporting hospitalized patients with enrollment into a health portal using a primarily student, volunteer-staffed model is feasible and acceptable to patients. Future research should focus on the impact of JeffDOT on patient outcomes and health behaviors.

## Introduction

While the COVID-19 pandemic accelerated the use of digital health technology out of necessity, it also highlighted an underlying digital divide threatening to exacerbate health disparities (1–5). Digital connectivity, as evidenced by more frequent patient portal use, telemedicine visits and digital communication with healthcare teams, and participation in online resources such as virtual education and support groups, can improve both patient and health system outcomes (6–9). Yet many individuals are unable to find and download a health app, log into a patient portal, or participate in a telehealth visit (1, 3, 10–13). In addition, many of these same individuals have low trust in the use of digital technology to receive health care, thus resulting in ongoing decreased use of digital health tools (1, 14–16). Digital health literacy levels are not always attributed to digital access, but instead can be more directly related to knowledge and trust of the digital resources available (17). It is critical to provide support to populations with lower use of digital health technology, as evidence suggests that use of digital health technology to manage health information and communicate with healthcare providers results in better health outcomes (9).

Significant funds have been invested across the U.S. over the past five years to increase access to technology and address digital literacy barriers, supporting initiatives such as community-based digital literacy classes. Yet many individuals still have insufficient skills to support use of digital health tools including use of the patient portal and telemedicine video visits (18, 19). One potential explanation is that resources such as scheduled community-based classes are not accessible to many people due to a variety of barriers including, but not limited to, lack of time available to commit to the class schedule, transportation needs, and childcare issues. A viable alternative to educating individuals in the community is approaching them while they are in the hospital, where access to education is not a barrier because it is brought to their bedside. Approaching hospitalized patients can make use of what is often extensive “down time” for patients while they wait for testing and for sufficient clinical improvement to be discharged. In addition, provision of education and support in digital health technologies while in the hospital capitalizes on this being a peak time of relevance to patients. Engaging patients while hospitalized opens the possibility for them to immediately apply their new skills in patient portal use to engage in their current care, as many portals (including the one used at the institution where this work took place) allow for patients to view real-time data during hospitalization including test results, names of their care team members, and their daily schedule. Yet to date, interventions focused on supporting patients with digital health onboarding while hospitalize are lacking.

To that end, our team developed the Jefferson Digital Onboarding Taskforce (JeffDOT). JeffDOT is a group of staff and students who approach patients while they are hospitalized to provide one-on-one teaching on how to sign up for and use the patient portal, with the goal of empowering patients to take a more active role in their health. The purpose of this paper is to describe implementation and preliminary outcomes of JeffDOT.

## Methods and materials

### Design, sample and setting

This retrospective descriptive study examined the implementation and preliminary outcomes of JeffDOT from August 22, 2023 through August 30, 2024. The JeffDOT team is based at Thomas Jefferson University in Philadelphia, PA. All patient outreach was conducted at three hospitals within the Jefferson Health system: Thomas Jefferson University Hospital (TJUH), Methodist Hospital, and Abington Hospital. This project was initially designed as a quality improvement project, and as such patient consent was not obtained. Study activities were approved by the Thomas Jefferson University Institutional Review Board.

### JeffDOT structure and process

JeffDOT is overseen by staff within the Jefferson Center for Connected Care, a university-based research center at Thomas Jefferson University. Most JeffDOT members are medical students at Sidney Kimmel Medical College within Thomas Jefferson University. In addition, a few health system community health workers and other volunteers have participated in the work of JeffDOT. Students and volunteers are recruited through word of mouth, student involvement fairs conducted within the Medical College, or referrals from staff or other students.

All JeffDOT members complete a structured onboarding process before participating in any patient interactions. This onboarding process was developed by Center for Connected Care staff and includes participation in a 30-minute virtual training led by a senior team member which outlines the purpose of the taskforce, what is expected of team members, and basic information of the process for completing JeffDOT shifts. After completion of the virtual training, members complete an observation of at least two JeffDOT shifts completed by another JeffDOT member. In addition, the team developed a procedure manual that is shared with all JeffDOT members to standardize the portal enrollment process that includes step by step instructions of the portal enrollment process.

At the start of each JeffDOT shift, a JeffDOT team member runs a real-time report from the Epic electronic medical record (EMR) to generate a list of all hospitalized patients within the hospital at which the JeffDOT shift is being worked who do not have an active MyChart patient portal account. This list can be sorted by patient zip code to facilitate identification of patients who live within priority zip codes which are known to experience greater health disparities. In addition, the list can be sorted by language, so that Spanish-speaking JeffDOT members can identify and approach Spanish-speaking patients.

All patients who were 18 years or older, English or Spanish speaking, currently admitted to the hospital, and did not have an active MyChart patient portal account were potentially eligible for approach. Patients were excluded if they were currently in police custody or incarcerated and/or medically unstable, psychologically impaired or intoxicated, or otherwise unable to provide informed consent as assessed by a project team member.

Patients were approached by a member of the JeffDOT team in their hospital room to discuss the patient portal and assess interest in receiving help to sign up for the portal. Interested patients were guided through portal enrollment and given education about portal features and how to use the portal at that time, or at a later time if patients asked for the team to return. For those patients who were not interested in assistance, reasons for declining assistance were collected.

### Data collection

After patients were enrolled in their portal and received portal education, participants completed a demographic survey and questions about digital health readiness (20), health literacy (21), and empowerment using the patient portal after receiving individualized support with portal enrollment. Digital health readiness was assessed using the following five survey items that were taken from the Digital Health Readiness Screener tool developed by our team: 1) “Do you have access to the internet?”, 2) “Do you have someone who could help you with technology when needed?”, 3) “Do you feel comfortable using the internet?”, 4) “Are you concerned that you won't get high quality care on a telehealth video visit?”, and 5) “Are you concerned about the privacy of your information when using technology for your healthcare?” (20) Health literacy was assessed using the 4-question BRIEF health literacy screener (22). Empowerment using the patient portal was assessed with the following question generated by the team: “On a scale of 1–5, where 1 is not at all empowered and 5 is very empowered, how empowered do you feel taking an active role in managing your health after talking with our team today?” Program documents were reviewed to describe implementation procedures and the characteristics of students and staff who assisted patients with enrolling in the patient portal. Feedback about training and overall implementation was obtained from JeffDOT volunteers via a brief open-ended survey.

### Data analysis

As this study was performed retrospectively and using data collected as part of a quality improvement initiative, data collection and analysis did not follow a structured evaluation framework and was entirely exploratory. Descriptive statistics were used to summarize the demographic characteristics of the participants as well as their responses to items on digital health readiness, health literacy, and empowerment. Because the data were collected after the patient was enrolled in their portal and received portal education, the direct impact of the intervention was not measured. Characteristics of the students and staff that comprise the JeffDOT were reviewed and summarized. JeffDOT student survey feedback was reviewed and informally summarized by the team.

## Results

The JeffDOT team approached 1,215 hospitalized patients between August 22, 2023 – August 30, 2024. Of these patients, 28% (343/1,215) were enrolled in the patient portal, 50% (605/1,215) declined to participate, 7% (88/1,215) were ineligible, 4% (45/1,215) were already enrolled in the patient portal or enrolled on their own, and 11% (134/1,215) requested assistance later and then were not available for follow up. Reasons for decline varied: 80% (481/605) stated they were not interested, 8% (46/605) reported not having internet at home, 3% (17/605) reported not feeling well, and 10% (58/605) responded “Other”. Some of the reasonings patients gave for “Other” that were conveyed by the enrolling staff included, but not limited to, not having a smartphone or computer, feeling capable of enrolling on their own, or being visually impaired. Of the 343 patients who enrolled in the portal, 49% (168/343were older than 55 years, 56% (193/343) were male, 34% (116/343) were Black, and 19% (66/343) spoke Spanish at home. Participant characteristics are shown in Table 1.

**Table 1:** Participant characteristics (*N* = 343).

Characteristic	*n* (%)
Age	
18–25	35 (10.2)
26–35	44 (12.8)
36–45	45 (13.1)
46–55	50 (14.6)
56–65	80 (23.3)
66+	88 (25.7)
Declined	1 (0.3)
Sex	
Female	146 (42.6)
Male	193 (56.3)
Other	1 (0.3)
Declined	3 (0.9)
Race	
White	135 (39.4)
Black	116 (33.8)
Asian	5 (1.4)
Hispanic/Latinx	81 (23.6)
Other	5 (1.4)
Declined	1 (0.3)
Language spoken at home	
English	270 (78.7)
Spanish	66 (19.2)
Other	6 (1.7)
Declined	1 (0.3)

### Health literacy and digital health readiness

Regarding health literacy, 27% (91/343) of patients noted that they were either somewhat, a little, or not at all confident when filling out medical forms in their preferred language. One-third of patients enrolled (114/343) reported that they always or often have someone help them read hospital materials in their preferred language. Twelve percent (54/343, 4 participants declined this question) reported they often or always have problems learning about their medical conditions because of difficulty understanding written information.

Of the 343 patients enrolled in their portal, almost all (94%) of patients reported having access to the internet though close to one quarter (22%) indicated that they do not feel comfortable using the internet. Most patients (84%) indicated that they have someone to help them with technology if needed. About one quarter of patients reported concern about the quality of care they receive during a telehealth video visit while 38% reported that they are concerned about their privacy when using technology for their healthcare. See Table 2.

**Table 2:** Health literacy and digital health readiness (*N* = 343).

Question	*n* (%)
1. How confident are you filling out medical forms in your preferred language by yourself?	
1 - Extremely	200 (58.3)
2 - Quite a bit	51 (14.9)
3 - Somewhat	49 (14.3)
4 - A little	20 (5.8)
5 - Not at all	22 (6.4)
Average score	***1.9***
Declined/Missed	1 (0.3)
2. How often do you have problems learning about your medical condition because of difficulty understanding written information in your preferred language?	
1 - Never	151 (44.0)
2 - Rarely	61 (17.8)
3 - Sometimes	73 (21.3)
4 - Often	28 (8.2)
5 - Always	26 (7.6)
Average Score	***2.2***
Declined/Missed	4 (1.2)
3. How often do you have someone like a family member, friend, hospital or clinic worker or caregiver, help you read hospital materials in your preferred language?	
1 - Never	118 (34.4)
2 - Rarely	39 (11.4)
3 - Sometimes	68 (19.8)
4 - Often	48 (14.0)
5 - Always	66 (19.2)
Average Score	***2.7***
Declined/Missed	4 (1.2)
4. How often do you have a problem understanding what is told to you about your medical condition?	
1 - Never	143 (41.7)
2 - Rarely	63 (18.4)
3 - Sometimes	55 (16.0)
4 - Often	26 (7.6)
5 - Always	15 (4.4)
Average Score	***2.02***
Declined/Missed^a^	41 (11.9)
5. Tech Access- Do you have access to the internet?	
Yes	322 (93.9)
No	17 (5.0)
Declined/Missed	4 (1.2)
6. Tech Knowledge - Do you have someone who could help you with technology when needed?	
Yes	287 (83.7)
No	53 (15.5)
Declined	3 (0.9)
7. Tech Knowledge - Do you feel comfortable using the Internet?	
Yes	263 (76.7)
No	76 (22.2)
Declined/Missed	4 (1.2)
8. Quality of Care - Are you concerned that you won't get high quality care on a telehealth video visit?	
Yes	94 (27.4)
No	241 (70.3)
Declined/Missed	8 (2.3)
9. Quality of Care - Are you concerned about the privacy of your information when using technology for your healthcare?	
Yes	131 (38.2)
No	207 (60.3)
Declined/Missed	5 (1.5)

The values in bold/italics represent the mean (average) response received to the corresponding question.

^a^ This question was added midway through data collection and was not asked of all participants. Thus, the number of missing responses is substantially higher.

### Post-Intervention empowerment and interest in bedside computer skills training

Sixty-four percent of the 343 enrolled patients reported that they felt very empowered to use the patient portal after speaking with a member of JeffDOT. Of the 343 enrolled patients, 45% expressed interest, with 27% of these being very interested when asked if they would be interested in additional basic computer skills training. See Table 3.

**Table 3:** Perceived empowerment and interest in bedside computer skills training (*N* = 343).

Response option	*n* (%)
Perception of empowerment after portal enrollment	
1 – Not at all empowered	7 (2.0)
2	12 (3.5)
3	34 (9.9)
4	68 (19.8)
5 – Very empowered	221 (64.4)
Declined/Missed	1 (0.3)
*Average*	*4.4*
Interest in bedside basic computer skills class	
1 – Not at all interested	189 (55.1)
2	9 (2.6)
3	30 (8.7)
4	22 (6.4)
5 – Very interested	92 (26.8)
Declined/Missed	1 (0.3)
*Average*	*2.5*

The value in italics represents the mean (average) response.

### JeffDOT implementation

The JeffDOT was comprised of 22 members including 16 medical students, 2 social work students, 1 community health worker and three staff members. From August 22, 2023 to August 30, 2024, the team worked a total of 216 shifts, with each shift lasting approximately two hours. The JeffDOT member survey feedback was overall limited, though suggested that students appreciated the structured training including direct observation that was provided for their onboarding and felt prepared to assist patients with portal enrollment.

## Discussion

We describe initial findings from implementation of a newly developed program (JeffDOT) designed to provide individualized digital literacy support to hospitalized patients. Our findings suggest that providing individualized support to hospitalized patients for enrollment into a health portal with a primarily student, volunteer-staffed model is feasible and acceptable to patients. Over one quarter of patients who were approached based on an EMR-generated list were successfully enrolled in the patient portal, with the majority of those who were enrolled (84%) reporting that they felt empowered (reported 4 or 5 on a scale of 1–5) to use the portal after receiving support from the JeffDOT team. Further, close to half reported that they would be very interested in additional basic computer skills training if offered by the hospital.

While prior studies have demonstrated that digital health technologies can improve outcomes such as treatment adherence and patient self-confidence (9, 23), differences in knowledge of and skills in using digital health technologies persist (17). The wide range of digital health readiness barriers alongside persistent disparities in health outcomes among low-income and minority racial populations suggest a need for interventions designed to identify and address each patient's unique barriers to digital health readiness with a focus on supporting these populations (20, 24–26). Recent work exploring barriers to digital health uptake among a Hispanic population in Philadelphia identified the need for individualized support to overcome many people's barriers to portal use (18), which is exactly what JeffDOT was designed to provide.

Privacy of healthcare information has received considerable attention given recent reports of data breaches within healthcare institutions and levels of mistrust among historically marginalized ethnic and racial populations (1, 15, 16). Thirty eight percent of the study sample reported that they are concerned about their privacy when using technology for their healthcare, which is similar to findings from a national study indicating that 36% of participants were “somewhat or very worried” about the privacy of their personal health information when shared with providers (9). Further 27% of the population engaged in this study expressed concerns about not receiving high quality healthcare via telehealth. While the technical aspects of connectivity for telehealth services are important, building a foundation of trust, confidence, and relevance of telehealth to patients' lives is also critical to support uptake patients' digital health readiness and ultimate uptake of telehealth (18, 20). This is especially true for patients with limited English proficiency navigating a primarily English-speaking healthcare system (10). Notably, 50% of the population approached declined assistance. While we do not have sufficient detail to further understand drivers of the decision to decline, we suspect this is at least in part due to trust and privacy concerns. As a result, we have made efforts to refine our approach to ensure patients understand the safeguards in place to protect their information and privacy.

The sustainability of an initiative such as JeffDOT requires a committed workforce. JeffDOT has been designed as a primarily volunteer-based initiative, with medical students conducting patient outreach and enrollment. The medical students who participated in JeffDOT found such value in this initiative as an opportunity for students to provide a direct service to patients during their initial medical school years that they have subsequently established JeffDOT as a formal student organization. The JeffDOT Student Organization, which was officially approved Summer 2024, meets regularly and actively recruits medical students to the organization as well as student leaders to ensure hand off of protocols from year to year. Establishing this organization facilitates sustainability of JeffDOT and could be replicated by other academic medical centers.

There are limitations to this study. The study took place in hospitals within the same health system located within Philadelphia, PA, thus, findings may not be generalizable to populations living in different geographic regions. In addition, this was designed as a patient service and not a research study, and as such data on outcomes beyond acceptability of portal enrollment are lacking. As the data collection took place only after the intervention, impact of the intervention could not be explicitly measured. We did not measure social desirability bias which may have influenced patients' ratings of empowerment after receiving support. We also do not have data on subsequent use of the portal by patients assisted by JeffDOT, thus limiting understanding of retention of information by patients and ultimate impact of this work on patient behaviors and outcomes. Further work is needed to assess the impact of JeffDOT on subsequent patient portal use and health outcomes. Despite these limitations, our study is novel and describes the design and implementation of a successful student-led digital on-boarding taskforce in an acute care hospital setting. This study contributes to a growing body of literature exploring best practices for a patient-centered approach that addresses challenges, barriers, and opportunities around digital health literacy and readiness in an underserved population (27).

In conclusion, we describe the successful implementation and outcomes of a primarily volunteer-based initiative designed to increase patient portal uptake among hospitalized patients. Activities were acceptable to patients and to our student volunteers, and findings suggest that there is need and desire among hospitalized patients for support in learning the skills needed to be proficient in use of technology both for general tasks (e.g., accessing email, navigating the internet) as well as accessing healthcare services (e.g., use of the patient portal). Next steps include expansion of this work to include provision of bedside digital literacy computer training as well as implementation of a longitudinal study to assess the impact of JeffDOT on patient outcomes.

## Data Availability

The original contributions presented in the study are included in the article/Supplementary Material, further inquiries can be directed to the corresponding author.
